# Sustainability Trait Modeling of Field-Grown Switchgrass (*Panicum virgatum*) Using UAV-Based Imagery

**DOI:** 10.3390/plants10122726

**Published:** 2021-12-11

**Authors:** Yaping Xu, Vivek Shrestha, Cristiano Piasecki, Benjamin Wolfe, Lance Hamilton, Reginald J. Millwood, Mitra Mazarei, Charles Neal Stewart

**Affiliations:** 1 Department of Plant Sciences, University of Tennessee, Knoxville, TN 37996, USA; yxu86@utk.edu (Y.X.); vshresth@utk.edu (V.S.); cristiano.piasecki@atsibrasil.com.br (C.P.); bwolfe@vols.utk.edu (B.W.); lhamil24@utk.edu (L.H.); 2 Center for Bioenergy Innovation, Oak Ridge National Laboratory, Oak Ridge, TN 37830, USA; 3 ATSI Brasil Pesquisa e Consultoria, Passo Fundo 99054-328, RS, Brazil

**Keywords:** sustainability, switchgrass, rust disease, chlorophyll, nitrogen, lignin, UAV, high throughput modeling

## Abstract

Unmanned aerial vehicles (UAVs) provide an intermediate scale of spatial and spectral data collection that yields increased accuracy and consistency in data collection for morphological and physiological traits than satellites and expanded flexibility and high-throughput compared to ground-based data collection. In this study, we used UAV-based remote sensing for automated phenotyping of field-grown switchgrass (*Panicum virgatum*), a leading bioenergy feedstock. Using vegetation indices calculated from a UAV-based multispectral camera, statistical models were developed for rust disease caused by *Puccinia novopanici*, leaf chlorophyll, nitrogen, and lignin contents. For the first time, UAV remote sensing technology was used to explore the potentials for multiple traits associated with sustainable production of switchgrass, and one statistical model was developed for each individual trait based on the statistical correlation between vegetation indices and the corresponding trait. Also, for the first time, lignin content was estimated in switchgrass shoots via UAV-based multispectral image analysis and statistical analysis. The UAV-based models were verified by ground-truthing via correlation analysis between the traits measured manually on the ground-based with UAV-based data. The normalized difference red edge (NDRE) vegetation index outperformed the normalized difference vegetation index (NDVI) for rust disease and nitrogen content, while NDVI performed better than NDRE for chlorophyll and lignin content. Overall, linear models were sufficient for rust disease and chlorophyll analysis, but for nitrogen and lignin contents, nonlinear models achieved better results. As the first comprehensive study to model switchgrass sustainability traits from UAV-based remote sensing, these results suggest that this methodology can be utilized for switchgrass high-throughput phenotyping in the field.

## 1. Introduction

Switchgrass (*Panicum virgatum*) is a perennial C4 grass that is widely considered as a leading candidate for bioenergy production. Its natural traits, including high biomass production, wide adaptation, and low agronomic input requirements, make it a highly desirable bioenergy feedstock. Increased productivity and sustainability of bioenergy plant feedstocks are key factors for biofuel production. Factors affecting switchgrass sustainability can be broadly attributed to plant genetics and the growing environment, signifying the importance of performing field studies for successful establishment and subsequent sustainability of feedstocks.

Various physiological and biochemical traits in switchgrass, such as chlorophyll, disease severity, nitrogen, and lignin affect the development of high-yielding feedstocks for producing cellulosic ethanol as the end goal. Resistance to diseases such as rust is an important sustainability trait of switchgrass. Rust disease is caused by *Puccinia novopanici* (formerly known as *Puccinia emaculata*), the most prevalent disease of switchgrass in the field, which significantly impacts yield and biomass quality [1,2]. Nitrogen use efficiency (NUE) is another important sustainability trait given the high cost of fertilization [3,4]. Low NUE and little fertilizer would decrease switchgrass biomass production [5,6,7,8,9,10]. Lignin is a secondary metabolite found in the cell walls of plants and a major contributor to the cell wall recalcitrance as it inhibits the accessibility of cellulose to enzymatic breakdown into fermentable sugars for biofuel production [11]. Nonetheless, the goal of lignin valorization is to transform lignin into value-added products such as low-cost carbon fibers and plastics [12]. Hence, switchgrass breeders might have to develop low or high lignin content genotypes based on the end-user goal. Switchgrass is an allopolyploid with very high heterozygosity. It is an obligate outcrosser, but can be clonally propagated. Thus, genetic improvement via traditional breeding is time consuming [13]. Moreover, phenotyping lignin content and composition in a destructive manner is another bottleneck. High throughput phenotyping of lignin content and its modeling and prediction have great potential to rapidly identify useful switchgrass accessions. These traits of interest are important for switchgrass sustainability. A scalable method for acquiring these traits is critical, especially for large spatial and temporal domains. The increasing demand for switchgrass sustainability assessment requires high throughput modeling of sustainability traits, cost-effective, yet reasonably accurate methodology, which can reduce the cost of labor and decrease time required.

Compared to morphological traits, such as plant height and leaf area index, chlorophyll and nitrogen contents are arduous to measure manually in the field [14,15]. These two physiological traits are challenging to assess by human eyes, but are detectable using a multispectral camera [16]. Mapping chlorophyll content has been performed for crops such as corn and wheat [17,18]. Studies show that nitrogen is positively correlated with chlorophyll content, and thus provides a scientific basis for predicting leaf nitrogen content. As a result, using unmanned aerial vehicle (UAV) images to explore the nitrogen content in plant leaves has gained significant attention in recent studies [19,20,21,22]. However, whereas remote sensing technology has been used extensively in many grain crops for phenotyping [17,21,23,24], it has not been used in switchgrass until recently [25]. For instance, methods for nitrogen content prediction for switchgrass is lacking [25,26] and high throughput lignin modeling has not been investigated in switchgrass and other bioenergy crops [27,28,29]. For rust disease, high throughput phenotyping has been mainly focused on wheat yellow rust disease [30,31,32], however, there is a gap in the literature for UAV data acquisition for rust diseases via high throughput modeling. Automated phenotyping of large field studies would be a significant advancement by saving time over traditional hand-acquired measurements as well as providing consistency in data acquisition.

Previously, we demonstrated high-throughput switchgrass phenotyping and biomass modeling by UAV in the field [25]. In the previous study, we determined that sustainable production of switchgrass required some genotypes that have efficient use of nitrogen, resistant to major pathogens (e.g., rust diseases), and tolerant to drought and other stresses. Factors that can be measured from physiological and productivity phenotyping, also play an important role in the yield stability of switchgrass [33]. Sustainable production of specialty biofuels and bioproducts from lignocellulosic feedstocks have great potential [34]. However, data for these traits have been collected manually in the field, without the application of UAV-based observations. Lignin, chlorophyll, nitrogen, and disease are particularly important for the development of high-yielding nitrogen-efficient switchgrass feedstocks for sustainable cellulosic ethanol production as an end goal. These traits are tightly associated with photosynthesis and biomass production, however, taking phenotypic data by hand is inefficient and not readily feasible except in very small populations. UAV-based methods allow scalability of efficiency.

In the present study, we expanded UAV-based remote sensing for enabling rapid sustainability trait data acquisition for the following: chlorophyll, rust disease severity, nitrogen, and lignin content in field-grown switchgrass. To develop an automated phenotyping approach, our objectives in this study were (1) to generate statistical models for rust disease severity and chlorophyll, nitrogen, and lignin contents using various vegetation indices, which were calculated from data collected by a UAV-based multispectral camera, and (2) to validate the UAV-based models by ground-truthing via correlation analysis between the traits measured manually with UAV-based data. To our knowledge, this is the first report on UAV-based high-throughput system for automated multi-trait assessments of field-grown switchgrass.

## 2. Results

### 2.1. Chlorophyll Content Measured on the Ground Compared with UAV Derived Vegetation Indices

**Figure 1:**
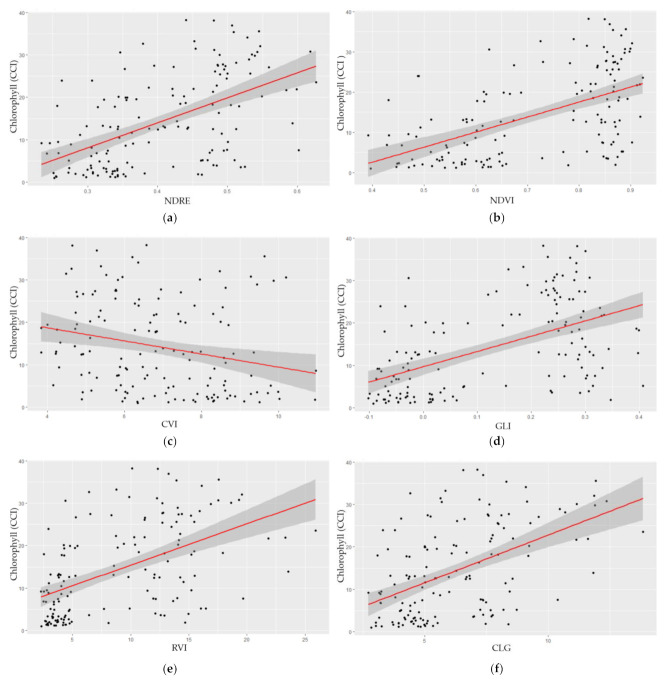

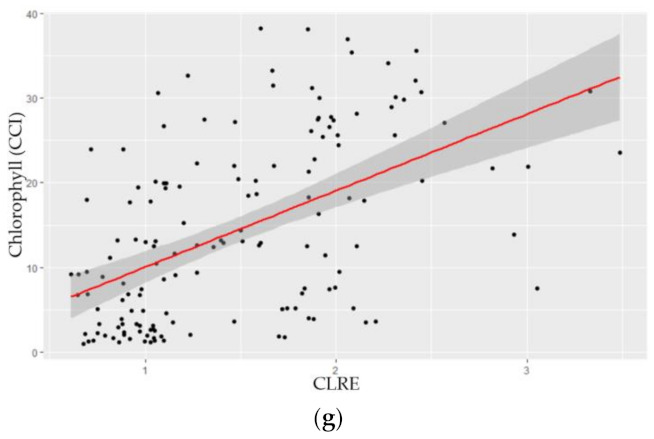
Sustainability trait models for chlorophyll content. Statistical models developed from UAV-based multispectral camera data and verified with ground-based data. (**a**) normalized difference red edge (NDRE); (**b**) normalized difference vegetation index (NDVI); (**c**) chlorophyll vegetation index (CVI); (**d**) green leaf index (GLI); (**e**) ratio vegetation index (RVI); (**f**) chlorophyll index green (CLG); (**g**) chlorophyll index red edge (CLRE). *X*-axis represents the vegetation index, and *y*-axis represents the chlorophyll content index (CCI) measured from the chlorophyll content meter, defined as CCI = % transmittance at 931 nm/% transmittance at 653 nm.

Seven vegetation indices, NDVI, NDRE, chlorophyll index green (CLG); chlorophyll index red edge (CLRE), chlorophyll vegetation index (CVI), green leaf index (GLI), and ratio vegetation index (RVI), commonly used for chlorophyll modeling, were tested for modeling chlorophyll content in switchgrass shoots (Table 1). NDVI resulted in a higher R-squared than NDRE, and the other five indices (Table 1 and Figure 1). Refer to the wave trough at ~625 nm that represents the chlorophyll absorption, a typical wavelength for the red band. NDVI uses the red band, rather than the red edge. When subtracted from the near infrared (NIR) band, the red band can create a more significant difference than the red edge band. Therefore, NDVI outperforms NDRE for modeling the chlorophyll content in the switchgrass. NDVI visually showed a similar data variation in the scatter plot (Figure 1a) as compared to NDRE (Figure 1b). Figure 1a shows that NDVI ranges between 0.40 and 0.90, whereas Figure 1b shows that NDRE has a lower range of values, from 0.25 to 0.6. However, Figure 1a shows a clustering effect when NDVI values are high. NDVI had lower homogeneity in the model (Figure S4). These features indicate that NDVI is may saturate under high vegetation density [34,35,36], whereas NDRE does not have this issue.

It should be noted that the Pearson correlation (r) between CVI and chlorophyll content was negative, whereas all the other six indices showed a positive correlation, although the Pearson correlation is weak (−0.2433). This can be explained from the index, in Equation (5), CVI = NIR × Red/(Green × Green), the green band is given a higher weight. Higher chlorophyll induces higher reflectance in the green band, rendering a lower CVI value.

### 2.2. Comparison between Rust Disease Severity Measured on the Ground and Vegetation Indices

**Figure 2:**
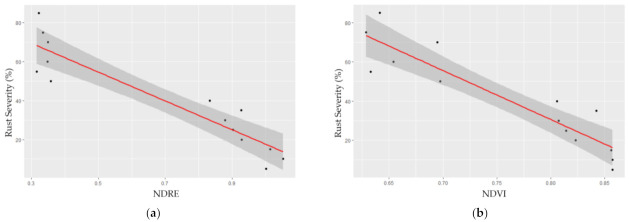
Model comparison between rust severity with (**a**) normalized difference red edge (NDRE) and (**b**) normalized difference vegetation index (NDVI). NDRE showed a slightly higher R-squared compared with NDVI. *X*-axis represents the vegetation index, and *y*-axis represents the rust severity measured on the ground.

The two common vegetation indices (NDRE and NDVI) for plant disease modeling and monitoring performed similarly with significant *p*-values. NDRE (R-squared = 0.8268) slightly outperformed NDVI (R-squared = 0.8285), with both models having significant *p*-values (2.82 × 10^−6^, and 3.82 × 10^−6^). Examining the index values, NDVI ranged between 0.65 and 0.85, whereas NDRE showed a wider range of values, from 0.3 to around 1 (Figure 2). This indicated that NDVI was oversaturated when the study area is highly vegetated [35,36,37], whereas NDRE did not have this issue.

Note that the rust severity was rated as a percentage of plant coverage with rust symptoms ranging from 0–100% where 0 indicates no rust and 100% indicates the whole plant is covered with rust (Figure S3). The rust severity was recorded every 5%, so 5%, 10%, 15%, up to 95% were recorded. In modeling, there was a statistical goodness of fit between rust disease severity and the vegetation indices.

### 2.3. Nitrogen Content Modeling: Challenges Present in the Results

NDRE and NDVI were used to model nitrogen. Table 2 shows the modeling results for mid-season and end-of-season. The results showed that the generalized additive model (GAM), a nonlinear model, slightly outperforms the other three models for both mid-season and end-of-season. However, the GAM model, as well as the three other models (linear regression, logarithm, and quadratic polynomial regression) were unable to predict the nitrogen content in the switchgrass. As shown in Table 2, for the end-of-season, NDRE was slightly more accurate compared to NDVI with the GAM model, as well as with the other three models. For mid-season, NDVI is apparently, slightly more accurate compared with NDRE, however, the *p*-value was not significant (0.1810). Therefore, compared with NDVI, NDRE generally achieved better modeling performance.

Comparing with the mid-season [R-squared 0.0187 (NDVI) and 0.0007 (NDRE)], the end-of-season had better modeling results to predict the nitrogen content [R-squared 0.0458 (NDVI) and 0.0521 (NDRE)] (Table 2). This result indicated that the high-throughput modeling approach benefited from the multispectral images when nitrogen content is lower in the tiller and leaves at the end-of-season.

**Figure 3:**
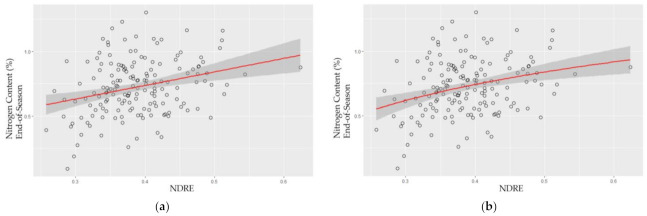

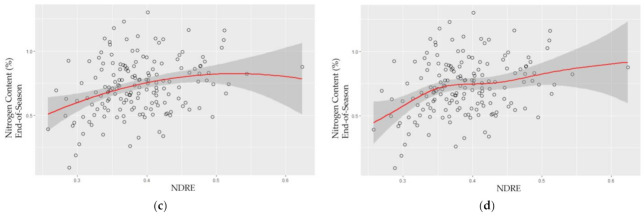
Nitrogen content model with normalized difference red edge (NDRE), (**a**) linear regression, (**b**) logarithm, (**c**) quadratic polynomial, vs. model (**d**) Generalized Additive Model (GAM). *X*-axis represents the vegetation index (NDRE), and *y*-axis represents N content (%) in tillers.

The models from NDRE are shown in Figure 3. The best model, GAM model is shown in Figure 3b, which still had a large variation in the model.

### 2.4. Polynomial Model Outperformed Other Models for Lignin Content Modeling

As shown in Table 3, the quadratic polynomial model performed the best for lignin content modeling. The three models (linear regression, logarithm, and quadratic polynomial) yielded similar results, whereas quadratic polynomial regression slightly outperformed other models (Table 3). Note that the *p*-values for the GAM models were above 0.1, which meant the nonlinear effect in the models was not significant. Table 3 also showed that end-of-season lignin content could be better predicted than the mid-season lignin content. NDVI slightly outperformed NDRE, nonetheless, the mid-season models all yielded poor performance.

Comparing the mid-season with the end-of-season, the end-of-season yielded better modeling results. As the lignin content typically increases toward the end-of-season, this indicated that the high throughput modeling approach with multispectral images benefited from relatively high lignin content in the tiller and leaves, which was in the opposite trend from the nitrogen modeling at the end-of-season.

**Figure 4:**
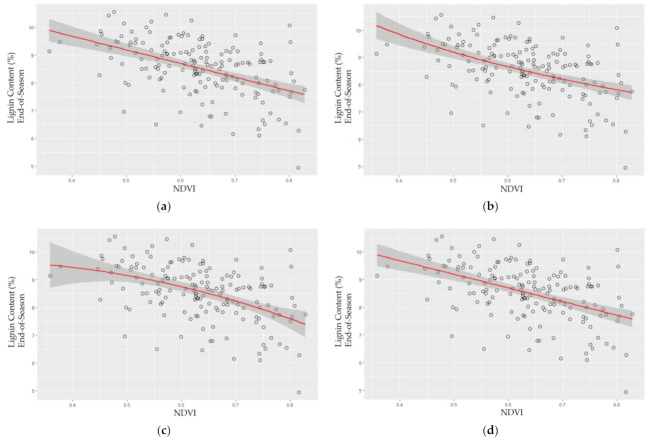
Lignin content model with normalized difference vegetation index (NDVI), (**a**) linear regression, (**b**) logarithm, (**c**) quadratic polynomial, (**d**) Generalized additive model (GAM) at the end-of-season. Note that (**d**) is very similar to (**a**) due to the strong nonlinear effect of the GAM model. *X*-axis represents the vegetation index (NDVI), and *y*-axis represents lignin content (%) as determined by near-infrared spectroscopy (NIRS) of field-collected tillers.

Figure 4 shows the lignin models from NDVI. The best model, the quadratic polynomial linear model, is shown in Figure 4c.

## 3. Discussion

Using a single UAV-based multispectral camera we developed a methodology for modeling rust severity as well as the contents of chlorophyll, nitrogen, and lignin. For the first time, lignin content was estimated in switchgrass shoots via UAV multispectral image analysis. However, there are several limitations, challenges, and opportunities of this study, which are discussed below. 

### 3.1. Model Accuracy

High throughput modeling for sustainability traits is an inherently challenging task. In the present study we achieved variable accuracy for four sustainability traits; rust disease (high accuracy; R-squared 0.83), leaf chlorophyll (moderate accuracy; Pearson correlation 0.54), nitrogen (very low accuracy, R-squared 0.05), and lignin content (low accuracy; R-squared 0.27). This variation in the accuracy was based on the use of different vegetation indices, the statistical models applied, and different sampling periods during of the growing season.

Apart from these factors, several other factors in the field of remote sensing may affect the quality of image analysis. Data acquisition may limit the availability of the best image quality used for analysis. For instance, model accuracy can be impacted by the accuracy of ground control points (GCPs) and reflectance calibration. GCPs are used to georeference and scale photogrammetric models and for camera calibration purposes. Typically, a minimum number of three GCPs is needed for small sites [38] and we applied between 5–10, well-distributed GCPs over the area for accurately georeferenced images. However, the georeferencing accuracy may introduce variation and large errors depending on the terrain, the difficulty of the area, and the variation in the above ground level [39,40]. In addition, ambient conditions on specific dates may cause calibration issues. When converting the raw imagery to reflectance, the calibration panel under changing ambient conditions, may cause unexpected errors to reflectance data generation and therefore add to the uncertainty of the vegetation indices. Moreover, most of the images for reflectance calibration purposes were recognized by Pix4D automatically, however, per Pix4D, the measurement from the radiometric target has an error of 5% (personal communication with Pix4D experts), this error contributes to the overall accuracy of the methodology.

### 3.2. Vegetation Index Sensitivity and Their Contribution to the Accuracy of the Methodology

The vegetation indices were calculated by a 10-cm diameter circle centered on each switchgrass plant canopy which may cause a small bias since plants do not have identical canopy sizes and shapes. Although we compared different diameter buffer sizes, which is a common approach used in the literature [41,42,43], this uniform size assumption remains a challenge for achieving the best accuracy. This fixed buffer zone design may affect specific traits, e.g., as the lignin content is expected to be more concentrated in the stems than in the leaves, choosing slightly larger sizes of these buffer zones may include more stems [44]. However, this may cause more bias to the chlorophyll content and rust severity because they are more concentrated in the leaves, therefore smaller-sized buffer zones are preferred to include fewer stems. To precisely measure the vegetation index for each switchgrass location, we would need to create the size information of each switchgrass plant; such information was not available from the collected ground-based data. This challenge can be addressed in future studies by using the exact perimeter for each switchgrass plant in the population.

With regards to chlorophyll modeling, we tested seven indices widely used in the literature [16,31,45,46,47,48]. While all seven indices performed similarly, the higher Pearson correlation and R-squared showed that NDVI and NDRE stand out from the other five indices. This finding is in line with the previous studies on sorghum [16] and maize [17]. This implies that for end-users in an agricultural or forestry setting, any of these indices can be adopted for chlorophyll modeling; however, for a long-term chlorophyll monitoring project, we recommend NDVI to be the best candidate for chlorophyll modeling because of its overall better performance.

From these findings, we refined our vegetation index set by focusing on the two vegetation indices (NDRE and NDVI) for rust disease, nitrogen content, and lignin content modeling. For rust disease modeling, although the results for NDVI and NDRE were both reasonably good, we recommend NDRE since our analysis showed that NDVI is oversaturated when the study area is highly vegetated (Figure 1a).

For rust disease modeling, this study has achieved reasonably high accuracy in the models, both NDRE and NDVI yielded high model accuracy although NDRE slightly outperformed NDVI. The good performance of NDVI was also reported in other crops, e.g., wheat [31], whereas the results from NDRE and NDVI were also in line with soybean rust disease [49]. Our results showed that NDVI was oversaturated when the study area is highly vegetated while NDRE did not have this issue. However, we have observed other diseases that partially mimic rust disease symptoms such as *Bipolaris* disease. We are working on collecting data from a more advanced sensor, the hyperspectral camera, and we expect to see a significant difference among rust and *Bipolaris* diseases.

The vegetation index sensitivity was vastly different for nitrogen and lignin content modeling compared with that of chlorophyll content and rust disease incidence. At the end-of-season, both of these statistics were very low, although NDRE outperformed NDVI slightly (R-squared 0.0521 vs. 0.0428). However, at mid-season, neither vegetation index was effective. This can be explained by the results from the chlorophyll content modeling. As it is reported that nitrogen content is positively correlated with chlorophyll content [50,51], NDVI is prone to oversaturate toward the end-of-season [35,36,37], which means that the sensitivity of the NDVI index will be affected, causing the accuracy to drop significantly.

For nitrogen content modeling, the end-of-season estimates outperformed the mid-season estimate. This indicated that the high-throughput modeling approach benefited from the multispectral images when nitrogen content in the tiller decreases towards the end of the growing season [52]. Compared with other studies on other crops (e.g., sorghum [16], wheat [21,47], and rice [20], our study on switchgrass nitrogen content modeling was poor. Nitrogen content models had very poor accuracy at mid-season, and as the best result, the GAM model, performed poorly at the end-of-season (R-squared 0.0521), and Figure 3 shows the models still have a large variation. While the model accuracy is subject to the factors related to data collection and vegetation index sensitivity, model overfitting problems associated with the GAM model also affect the model accuracy as reported in Adamec et al. for a forest study [53]. High-throughput modeling of nitrogen content continues to be challenging with the current model and sensor technology, and due to this reason, we do not recommend this methodology for high throughput modeling with the UAV-based multispectral datasets with the current technology.

Lignin content in switchgrass leaves and stems increased toward the end-of-season [54]. Compared to the mid-season, a higher sensitivity of both indices toward the end-of-season was observed. This indicated the modeling approach worked well when there was relatively high lignin content in switchgrass tillers. NDVI outperformed NDRE slightly (R-squared 0.2727 vs. 0.1905). As far as we know, this is the first study to use UAV-based sensors to measure lignin content in plants. Given the importance of lignin content for switchgrass cultivar development, we believe the accuracy of this new approach is acceptable as a baseline for field-scale lignin phenotyping, but additional research is warranted to refined sensor development and deployment for this important cell wall component. Furthermore, applications of this methodology are recommended for end-of-season analysis in the current phase as mid-season lignin model accuracy is not yet satisfactory. Field-scale lignin modeling remains a challenge and needs to be addressed in future research.

### 3.3. Practical Applications for Agriculture and Forestry

As a comprehensive study on high throughput sustainability traits modeling, one objective is to reduce the labor and time costs inherent to ground-based phenotyping. It is important to transfer this methodology to practical applications. UAV-based techniques for bioenergy crop sustainability traits assessments are non-destructive and have significant applications in bioenergy crop breeding [55]. The UAV-based automated phenotyping approaches can facilitate the identification of superior genotypes and subsequently beneficial gene targets in a genome-wide association panel and choosing plants to use in breeding programs. This is more important for taller bioenergy crops such as poplar trees. Taller plants make manual phenotyping slow and challenging [56] and thus becomes a bottleneck for genotype selection studies [57]. Therefore, automated phenotyping, specifically to assess the sustainability traits, will be a significant advance in bioenergy crop studies.

Recent studies have shown that there is an increasing demand for high-throughput phenotyping in the agriculture sector [58]. UAV-based remote sensing holds great promise as a high-throughput phenotyping tool and this study could be applicable to other crops in agriculture sectors as well as in forestry [59]. The benefit and values brought by the UAV-based phenotyping is a matter of the spatial scales of the farm we are applying this methodology to: for small farmlands that are about the similar scale of the ETREC, manual phenotyping, including leaf sampling, destructive analysis, field-based traits measurements took typically several days or even weeks with more demanding on manpower than the UAV-based measurements; for commercial farmlands with several hectares, manual phenotyping is barely possible, and the UAV-based approach becomes an essential requirement and can be less costly and much faster compared with manual phenotyping for precision agriculture and plant breeding [60,61].

## 4. Materials and Methods

### 4.1. Data Collection and Processing Overview

#### 4.1.1. Data Collection and Processing Pipeline

**Figure 5:**
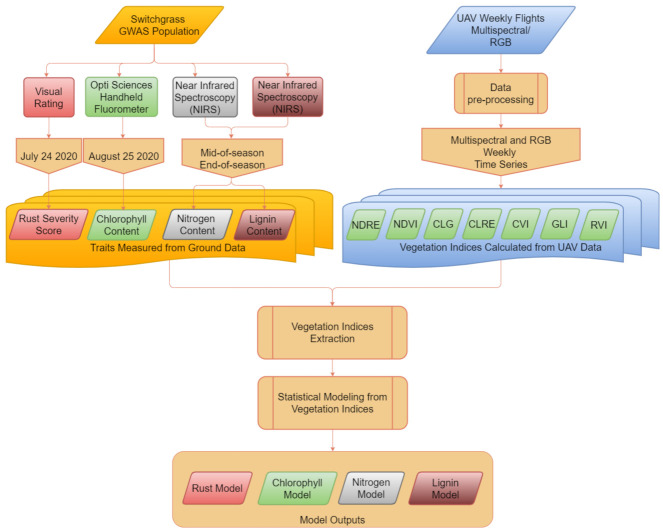
Data processing and analysis pipeline for switchgrass sustainability traits modeling.

The entire airborne data processing pipeline consisted of three sections: ground data collection, UAV data collection and processing, and sustainability trait modeling (Figure 5). More details are included in the following sections.

#### 4.1.2. Field Design and Ground Data Collection

**Figure 6:**
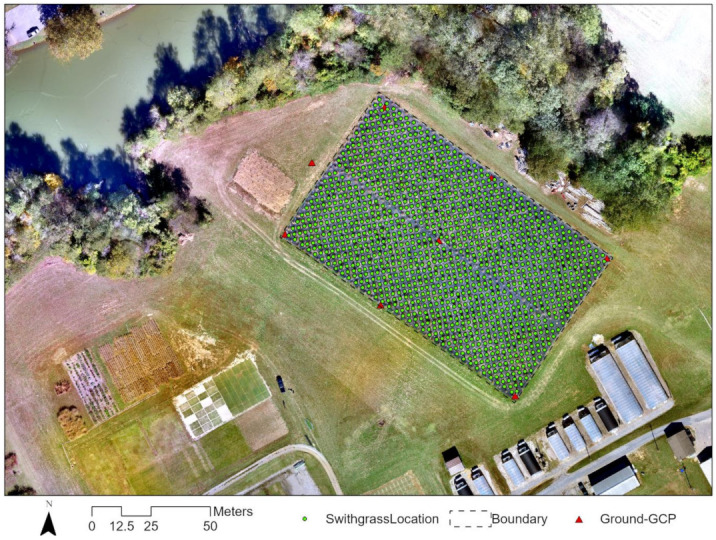
Field map of the switchgrass locations and ground control points (GCP). The color map is the composite of the R, G, and B bands collected with the Zenmuse camera. A total of eight control points (including one ground station), as shown in red triangles, were surveyed in the field. The Global Positioning System (GPS) antenna was manufactured by CHC Navigation (Shanghai Huace Navigation Technology Ltd., Shanghai, China). It is a static GPS, post-processing used Online Positioning User Service (OPUS) to resolve the high-accuracy positioning. The eight control points were collected in the field from 5 to 30 September 2019. The field ground- and UAV-based data for the four sustainability traits were collected at several time points (Table 4). Data collection for chlorophyll was at one time-point (mid-season), for rust disease at one time-point (mid-season), and for nitrogen and lignin at two time-points (mid-season and end of the season).

A collection of 330 lowland switchgrass accessions was planted in a field at the University of Tennessee Plant Sciences unit of the East Tennessee Research and Education Center (ETREC) in 2019 (Figure 6). The details of the plantation and experimental design were described in Li et al. (2020). The 330 accessions were planted under two nitrogen (N) fertility treatments: moderate (135 kg of N ha^–1^) and low (0 kg of N ha^–1^) nitrogen supplementation for a genome-wide association study. Each accession has four replicates in the field (2 replicates per N treatment), totaling 1320 switchgrass plants, plus 40 control plants (‘Alamo’ AP13), which were arranged in honeycomb design (Figure S1) with 2.8 m interplant spacing.

The chlorophyll content of the flag leaves was measured by an Opti-Sciences (Opti-Sciences Inc., Hudson, NH, USA) handheld chlorophyll content meter (model CCM-200 plus) during the 2020 growing season (year-2). For each plant, we sampled two leaves and calculated the average for a reliable reading.

The rust disease severity was evaluated by a visual rating system. AP13 control genotype is known to be highly susceptible to rust, thus, if the environmental conditions are in favor of the disease development, we were able to observe the initial symptoms of rust on AP13 plants. Once rust disease was estimated to be present in about 10% of the plants, the rust severity evaluation was started and performed every 14 days. The data obtained from 24 July 2020 (Table 4) was used for subsequent analyses. Rust severity was scored as the percentage of plant coverage with rust disease. The first symptoms of switchgrass rust began as very small brown or brick-red spots on leaves. The rust severity was rated as a percentage of plant coverage with rust symptoms ranging from 0–100% where 0 indicates no rust and 100% indicates the whole plant is covered with rust disease (Figure S3). The percentage of plant coverage with rust was estimated on the whole plant basis and recorded manually as rust severity for each plant, and every 5%, so 5%, 10%, 15%, up to 95% were recorded. 

The nitrogen and lignin content of the aboveground biomass were measured via near-infrared spectroscopy (NIRS) (FOSS 6500, Foss Nirsystems, Silver Spring, MD, USA) [62]. Two tillers containing stem and leaves were collected from each plant at two time points (mid-season and end-of-season) and the samples were oven-dried at 45 °C for 72 h. The dried tillers were then chipped into 5–8 pieces before milling. The chipped materials were milled with a Wiley mill (Thomas Scientific, Model 4, Swedesboro, NJ, USA) through a 20-mesh screen (1.0 mm particle size).

#### 4.1.3. UAV Data Collection

Plant UAV-based data were collected every week. For each weekly flight, the UAV team calibrated the flight systems (GPS and Daylight Sensors) in the field (Figures S1 and S2b), then executed the flight missions using programmed flight modes and completed the initial data collection from the field.

The UAV that carried the flight missions is Matrice 600 Pro (Figure S2), a professional level hexacopter manufactured by DJI (SZ DJI Technology Co., Ltd., Shenzhen, China). Two cameras were used to collect images during flights. MicaSense RedEdge Multispectral Camera (MicaSense, Inc., Seattle, WA, USA) is a five-band multispectral camera (Table 5). When flying at an above ground level (AGL) of 20 m, the best spatial resolution is 1.4 cm. ZenMuse X3 RGB Camera (SZ DJI Technology Co., Ltd., Shenzhen, China) is a three-band, higher resolution camera, with 0.91 cm resolution at 20 m AGL.

### 4.2. UAV Data Processing

The UAV data were collected with Pix4DCapture, and processing was performed in Pix4DMapper with agriculture multispectral mode enabled. An overview of the data pipeline:

UAV data collections on or around the dates for ground data sampling;

Pre-processed the MicaSense RedEdge multispectral data, created the orthomosaic imagery;

Calculated the normalized vegetation index (NDVI), normalized difference red edge (NDRE), and other five vegetation indices (Table 6);

We created a 10 cm (in diameter) circle above each switchgrass center using GIS software, and calculated the mean NDVI, mean NDRE, and other vegetation index average values for each circle; more explanation of this diameter selection is described later in Section 4.3.3.

Pixel sample size: for the entire switchgrass population locations, each location had 30 to 34 pixels to calculate the average vegetation index values. This pixel sample size is statistically appropriate for the calculation.

Analysis: comparing the modeling results derived from the seven vegetation indices with the field chlorophyll, rust severity, nitrogen, and lignin ratings, Pearson correlations and *p*-values were calculated, and linear equations were given. Nonlinear models were developed where linear models do not perform well.

#### 4.2.1. Georeferencing and Mosaicking

The raw data collected from a UAV does not contain accurate location information. Georeferencing used eight GCPs to optimize the location accuracy of the images. The images were then stitched together in Pix4DMapper to create an orthomosaic imagery, with which an accurate measurement is possible. Figure 6 shows the location of the GCPs.

#### 4.2.2. Reflectance Calculation

The calibration panel was used to convert the raw data to reflectance. These data were imported and processed in Pix4D. The albedo values of the panel, which is a measure of the amount of incident to the surface that is reflected without being absorbed, were pre-calculated by the manufacture based on the reflectance values they interpolated directly from the panel itself. Pix4D enables a quick response code (QR code) mode to accelerate workflows. The QR code contains characters representing two ends of a spectral line, and because the calibration panel has a very flat response across the wavelengths, a linear best fit of the reflectance curve between 400 nm and 1000 nm was calculated from the two ends of the spectral line. Most of the QR code images were recognized by Pix4D automatically, however, under some circumstances, the QR codes were not read correctly and hence the reflectance values were manually inputted into the calibration procedure.

### 4.3. Data Analysis

#### 4.3.1. Rationale

**Figure 7:**
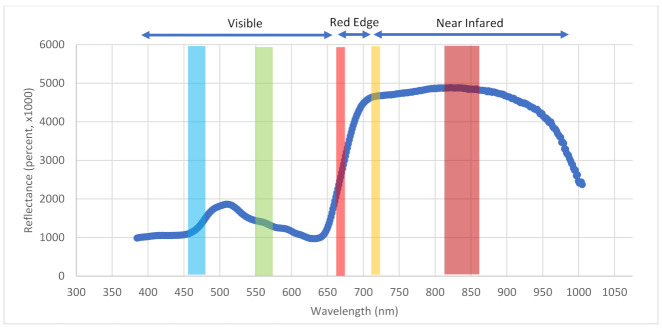
Spectral responses for healthy switchgrass leaves (collected from the Resonon Pika XC2 hyperspectral camera). The five ribbons, from blue in the left, to red in the right, indicate the band locations of the MicaSense multispectral camera, as compared to the full spectral range of Pika XC2.

To explain how to model the sustainability traits from the multispectral camera, we mapped the spectral responses for healthy switchgrass (Figure 7). The spectral response was created with a hyperspectral camera to show a smoother and higher spectral resolution curve. The spectral response from our Resonon XC2 hyperspectral camera in the switchgrass field showed that the near-infrared region appears to be very high in reflectance, which correlates with the high chlorophyll content in the leaves (Figure 7). The spectral curve also showed a peak around the 500 nm wavelength, between the green and the blue bands. These spectral features set the scientific ground for identifying sustainable traits from UAV-based cameras. As shown in Figure 7, the blue, green, and red lines mark the blue, green, and red band locations of the MicaSense multispectral camera in the full wavelength of the spectra. The gray line indicates the division of visible from near-infrared. The wavelength between the red and gray lines is commonly referred to as “red edge”. The spectral response was relatively flat in the near-infrared, and also a local peak in the visible between 500 nm and 525 nm wavelengths. The wave troughs at ~450 nm and ~625 nm represent chlorophyll absorption.

#### 4.3.2. Remote Sensing Indices

We calculated seven indices (Table 6) from the processed reflectance. These indices are associated with the five multispectral bands, commonly used for biomass, chlorophyll, nitrogen phenotyping [16,45,46,47]., and disease monitoring [31,48].

NDRE = (*NIR* − *Red Edge*)/(*NIR* + *Red Edge*) (1)

NDVI = (*NIR* − *RED*)/(*NIR* + *RED*) (2)

CLGreen = *NIR*/*Green* − 1 (3)

CLRedEdge = *NIR*/*Red Edge* − 1 (4)

CVI = *NIR* × *Red*/(*Green* × *Green*) (5)

GLI = (2 × *Green* − *Red* − *Blue*)/(2 × *Green* + *Red* + *Blue*) (6)

RVI = *NIR*/*Red* (7)

#### 4.3.3. Vegetation Indices Statistics

To extract the information for switchgrass sustainability traits, statistics were needed to draw from the region of interest on the images. The unit for the images, calculated for vegetation indices, were represented in pixel, which is 1.4 cm, identical to that of the multispectral images. However, a typical crown, or canopy, of the switchgrass, varied between 0.35 to 2 m. Apparently, the size of one pixel could not represent the area of the switchgrass canopy. Hence, we needed a larger scale to properly collect the statistics for the images, and we used a spatial analysis approach, the buffer zone approach, to achieve this goal [63,64].

**Figure 8:**
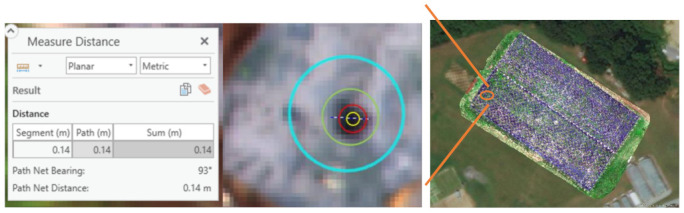
Comparison between 40 cm (blue), 20 cm (green), 10 cm (red), and 5 cm (yellow) buffer size over a single plant. Minimal canopy size in the field was ~14 cm (represented by the dark green footprint underneath the four circles).

The size of the buffer zone matters because it is the baseline for obtaining precise statistics. In the experimental design, we compared three different buffer zone sizes for the best summarize of the images that represent the switchgrass canopy: 40 cm, 20 cm, and 10 cm diameter circles (Figure 8). A buffer size of 10 cm diameter was chosen after checking the circumference of the switchgrass. Note that our field consisted of 1360 switchgrass plants. Instead of drawing the actual size of each switchgrass plant, we used a fixed size for all plants to represent the center of each plant canopy.

Differences among these three sizes had a significant impact on the statistics (Table 7). As the footprint of the switchgrass on UAV remote sensing images is a mixture of switchgrass and soil (and sometimes weeds were also included), choosing the proper buffer zone size is critical for obtaining accurate statistics from vegetation indices. To this end, we comprehensively tested these four sizes, and found that the diameter of 40 cm was over-sampled as not only switchgrass but also soils on the ground were included. We decided to reduce the size of the buffer zone to cover less area of the image but maintain a sufficient sample size to get an unbiased summary from the pixels. We found that when the diameter was reduced to 10 cm, sufficient samples representing the center of each switchgrass canopy were properly represented for each switchgrass plant.

**Figure 9:**
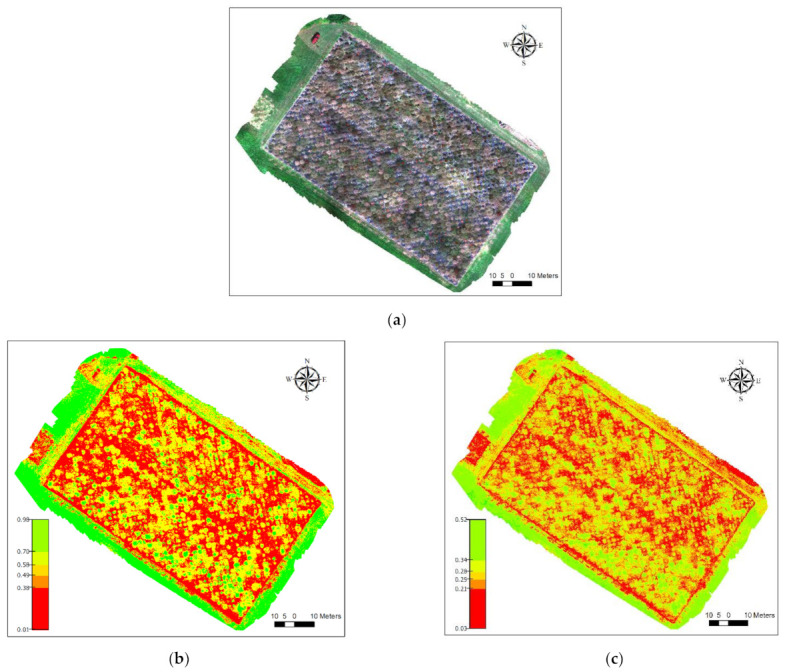
RedEdge bands (RGB) composite overlay with ground data. The green color of the two vegetation index maps represents higher leaf chlorophyll content compared to the red color that represents lower content of chlorophyll in leaves. (**a**) Map of the switchgrass field experiment made with the multispectral camera. (**b**) normalized difference vegetation index (NDVI) vegetation index map of the study area. (**c**) normalized difference red edge (NDRE) vegetation index map of the study area.

From the buffer zone, we obtained the statistics for the seven vegetation indices, and the ground-based data were overlayed with the UAV-based vegetation indices for statistical modeling. Figure 9 shows an example of how these two types of datasets were integrated into the study. The color map shown in Figure 9a is the RGB composite of the data collected with the MicaSense RedEdge-M camera. The ground-based data, e.g., rust severity, were converted into a GIS file, and georeferenced with the NDVI and NDRE vegetation index maps (Figure 9b,c). The green color of the two vegetation index maps represents higher leaf chlorophyll content compared to the red color that represents lower content of chlorophyll in leaves. Red-filled circles show the locations of the switchgrasses measured for chlorophyll content.

#### 4.3.4. Model Selection and Evaluation

**Figure 10:**
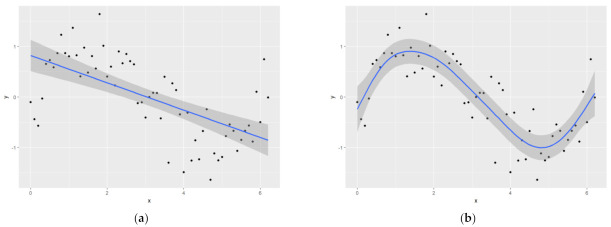
GAM model explained with comparison to regression, (**a**) linear regression; (**b**) generalized additive model (GAM).

Both linear and nonlinear models were built for chlorophyll, rust severity, as well as nitrogen and lignin contents. We started from linear regression analysis for these four sustainability traits, and a nonlinear model was considered for traits that cannot be explained with linear regression. Some data are best described by “wiggly models,” and one of the widely-used models is the generalized additive model (GAM, Figure 10) while simple linear models do not fit well. In recent years, GAM [65] found its application in remote sensing for crop sustainability studies [66], plant science [67,68], plant nitrogen [69] and lignin [70], biomass modeling [71], and other areas [72], and has great potential for switchgrass and bioenergy species [69].

We used root mean square error (RMSE) and Pearson correlation (r), and R-squared to assess the accuracy of the models. We split the data into training set and testing set to test the accuracy of the GAM model.

(8) $$\mathrm{RMSE}=\sqrt{\frac{\sum_{i=1}^{N}{\left(\hat{y_{i}}-y_{i}\right)}^{2}}{N}}$$

$y_{i}$ is the observed values of the *y* variable, $\hat{y_{i}}$ is the predicted value of $y_{i}$.

(9) $$r\;=\;\frac{\sum \left(x_{i}-\bar{x}\right)\left(y_{i}-\bar{y}\right)}{\sqrt{\sum {\left(x_{i}-\bar{x}\right)}^{2}\sum {\left(y_{i}-\bar{y}\right)}^{2}}}$$

$x_{i}$ is the *x* variable in the sample, $y_{i}$ is the y variable in the sample. $\bar{x}$ and $\bar{y}$ are the mean of the values of the *x* and *y* variables, individually.

(10) $$R-\mathrm{squared}=\frac{\sum {\left(\hat{y_{i}}-\bar{y}\right)}^{2}}{\sum {\left(y_{i}-\bar{y}\right)}^{2}}$$

$\bar{y}$ is the mean of y variable, $y_{i}$ is the observed values of *y* variable, $\hat{y_{i}}$ is the predicted value of $y_{i}$.

## 5. Conclusions

Using vegetation indices calculated from a UAV-based multispectral camera, our study developed statistical models for estimating leaf chlorophyll, rust disease, nitrogen, and lignin content. For chlorophyll modeling, NDVI performed better than NDRE and the other five indices, NDRE had less variance in the model compared to the NDVI model. For rust, NDRE was negatively correlated with rust severity and NDRE outperformed NDVI and therefore it may be generally recommended for rust disease modeling. Lignin content could be predicted using NDVI for the end-of-season estimates, and NDVI had better predictability for lignin than NDRE. Nitrogen content could not be predicted using NDVI for the end-of-season, however, NDRE had slightly better predictability for nitrogen content compared with NDVI. Overall, for a high throughput modeling study, linear models achieved satisfactory performances for rust disease and chlorophyll, and the polynomial model achieved the best performance for lignin content. However, modeling for nitrogen content is a challenge. Although the GAM model outperformed the other three models, the performance of modeling the nitrogen content for switchgrass needs further improvement.

## Figures and Tables

**Table 1:** Chlorophyll model performance. Seven vegetation indices were developed to model the chlorophyll content. All models are statistically significant. The seven indices are: normalized difference red edge (NDRE), normalized difference vegetation index (NDVI), chlorophyll vegetation index (CVI), green leaf index (GLI), ratio vegetation index (RVI), chlorophyll index green (CLG), and chlorophyll index red edge (CLRE).

VI	NDRE	NDVI	CVI	GLI	RVI	CLG	CLRE
Pearson Correlation	0.5386	0.5466	−0.2433	0.5225	0.5154	0.4975	0.5219
R-squared	0.2852	0.2939	0.0527	0.2681	0.2606	0.2424	0.2674
*p*-value	1.664 × 10^−12^	6.659 × 10^−13^	0.0029	9.628 × 10^−12^	2.041 × 10^−11^	1.249 × 10^−10^	1.028 × 10^−11^

**Table 2:** Linear models compared with nonlinear models for nitrogen content modeling with normalized difference red edge (NDRE) and normalized difference vegetation index (NDVI). Results showed both mid-season and end-of-season. *p*-values with * are significant.

		Linear Regression		Logarithm Transformation		Quadratic Polynomial		Generalized Additive Model (GAM) Model (Nonlinear Effects)		
		R-Squared	*p*-Value	R-Squared	*p*-Value	R-Squared	*p*-Value	R-Squared	*p*-Value	RMSE
Mid-season	NDVI	0.0020	0.3735	0.0025	0.3246	0.0083	0.3576	0.0187	0.1810	0.2466
	NDRE	0.0038	0.2284	0.0027	0.3085	0.0130	0.1684	0.0007	0.0281 *	0.2483
End-of-season	NDVI	0.013	0.0797	0.0111	0.0131 *	0.0299	0.0008 *	0.0458	0.0231 *	0.2130
	NDRE	0.0512	2.12 × 10^−7^ *	0.0190	0.0011 *	0.0306	0.0007 *	0.0521	0.0110 *	0.2117

**Table 3:** Linear model vs. nonlinear model for lignin. Results show both mid-season and end-of-season. Normalized difference red edge (NDRE) and normalized difference vegetation index (NDVI) were used for the modeling. *p*-values with * are significant.

		Linear Regression		Logarithm Transformation		Quadratic Polynomial		Generalized Additive Model (GAM) Model (Linear and Nonlinear Effects)			
		R-Squared	*p*-Value	R-Squared	*p*-Value	R-Squared	*p*-Value	R-Squared	RMSE	*p*-Value Linear Effect	*p*-Value Nonlinear Effect
Mid-season	NDVI	0.0319	0.0002 *	0.0306	0.0003 *	0.0368	0.0015 *	0.0092	0.8089	0.0001 *	0.0002 *
	NDRE	0.0433	2.00 × 10^−5^ *	0.0421	2.65 × 10^−5^ *	0.0587	1.73 × 10^−5^ *	0.0223	0.7988	1.8 × 10^−5^ *	0.0558
End-of-season	NDVI	0.2725	2.2 × 10^−16^ *	0.2686	2.2 × 10^−16^ *	0.2727	2.2 × 10^−16^ *	0.2329	0.8730	2.2 × 10^−16^ *	0.9414
	NDRE	0.1839	2.2 × 10^−1 6^*	0.1888	2.2 × 10^−16^ *	0.1905	2.2 × 10^−16^ *	0.1621	0.9122	2.2 × 10^−16^ *	0.1502

**Table 4:** Ground-based and UAV-based data collection for chlorophyll, rust, nitrogen, and lignin content (2020 growing season, year-2).

	Dates of Ground-Based Data	Dates of UAV-Based Data
Chlorophyll	25 August 2020	26 August 2020
Rust Disease	24 July 2020	29 July 2020
Nitrogen and Lignin	4 August 2020	29 July 2020
	10 November 2020	5 November 2020

**Table 5:** Specification of the multispectral camera.

Band Number	Band Name	Wavelength (nm)
1	Blue	465–485
2	Green	550–570
3	Red	663–673
4	Near Infrared	820–860
5	Red Edge	712–722

**Table 6:** Vegetation indices adopted in this study with their reference.

Vegetation Index	Definition	Commonly Used for
NDRE	normalized difference red edge	chlorophyll, nitrogen, disease
NDVI	normalized vegetation index	vegetation cover, chlorophyll, disease
CLG	chlorophyll index green	chlorophyll
CLRE	chlorophyll index red edge	chlorophyll
CVI	chlorophyll vegetation index	chlorophyll
GLI	green leaf index	greenness
RVI	ratio vegetation index	leaf area index, biomass

**Table 7:** Buffer zone sizes and their impact on the image analysis.

Buffer Zone Size (Diameter)	Sample Size (Pixels)	Impact
40 cm	480–640	Oversampled, soil included
20 cm	120–160	Oversampled, soil included
10 cm	30–40	Sufficient samples representing the proper area size of the switchgrass canopy.
5 cm	7–10	Insufficient samples due to under-sampled buffer zone size

## Data Availability

The raw data supporting the conclusions of this article will be made available by request to the corresponding authors.

## Supplementary Materials

The following are available online at https://www.mdpi.com/article/10.3390/plants10122726/s1, Figure S1. The field layout for the switchgrass nitrogen study. Figure S2. UAV system with the multispectral camera. Figure S3. Visual rating of rust disease in the switchgrass field. Figure S4. Residual plots as a comparison between the NDVI model and NDRE model for chlorophyll content.
