# Supplementary material for: Sustainability Trait Modeling of Field-Grown Switchgrass (Panicum virgatum) Using UAV-Based Imagery
Source: Plants (Basel). 2021 Dec 11;10(12):2726. doi: 10.3390/plants10122726 (PMC8709265; doi:10.3390/plants10122726)
Supplement: Supplementary file 1 [file plants-10-02726-s001.zip › plants-1472643-supplementary.pdf]

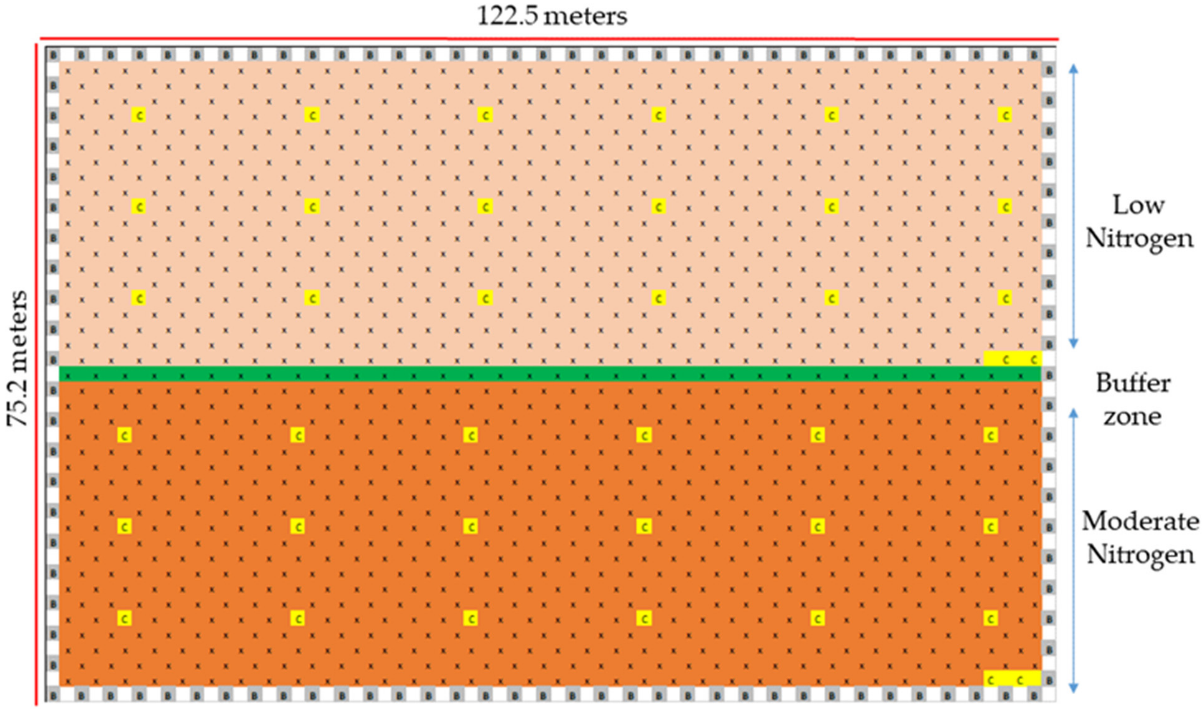

Figure S1. The field layout for the switchgrass nitrogen study. The field design includes 330 accessions (2 replicates per nitrogen treatment), 'Alamo' AP13 controls, 'Blackwell' buffer zone, and 'Blackwell' border with 2.8 m interplant spacing. The field site also includes weed cloth boundary. The field covered 0.92 hectares.

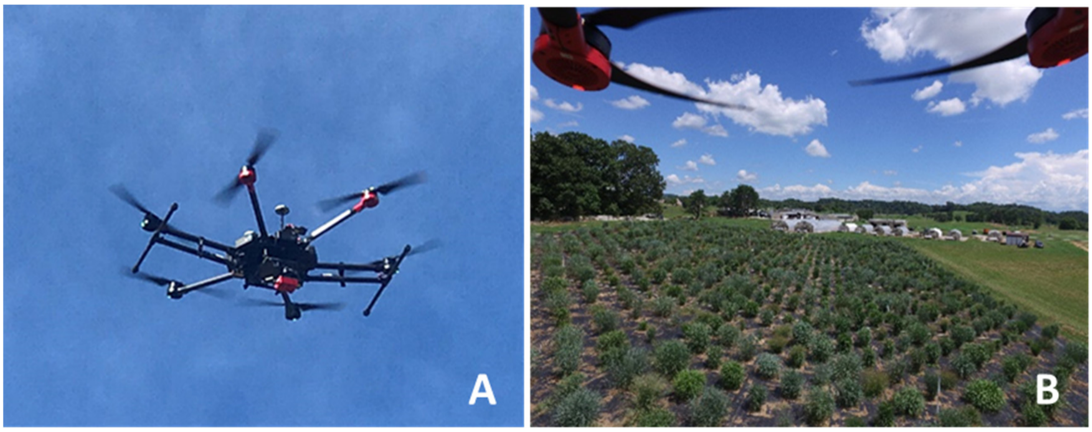

Figure S2. UAV system with the multispectral camera. (**A**) DJI Matrice 600 Pro with MicaSense RedEdge-M multispectral camera. (**B**) UAV system in a flight mission over switchgrass field site.

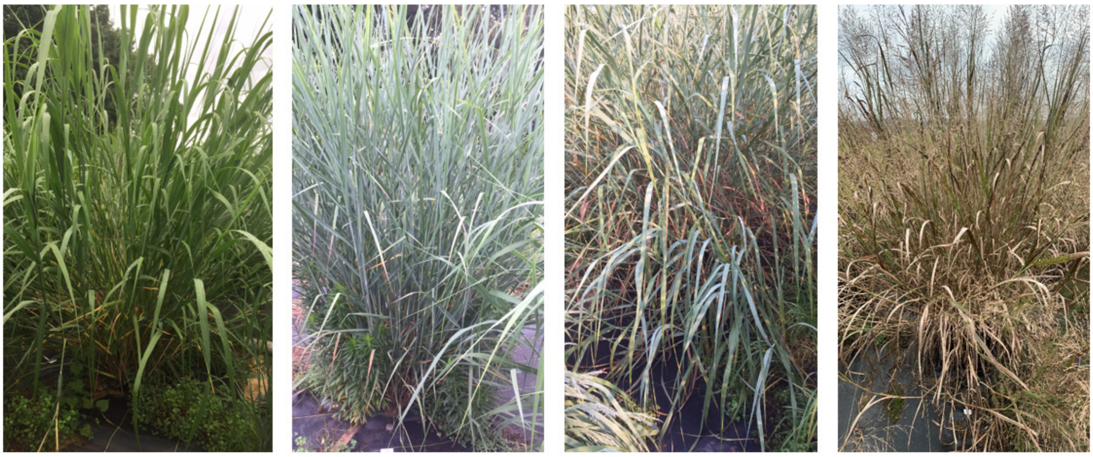

(a) <5% (b) 25% (c) 50% (d) 100%

Figure S3. The rust disease (*Puccinia novopanici*) severity of the switchgrass population was evaluated by a visual rating system, (**a**) less than 5%; (**b**) 25%; (**c**) 50%; (**d**) 100%. Less than 5% is when no rust disease or the first sign of rust disease emerges. 100% is the whole plant is infected by rust disease.

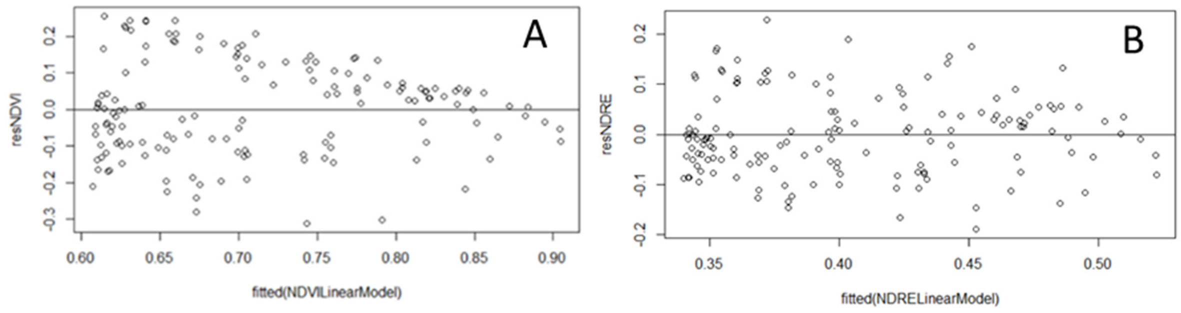

Figure S4. Residual plots as a comparison between the NDVI model and NDRE model for chlorophyll content. (**A**) residual plot for the NDVI model; (**B**) residual plot for the NDRE model. NDVI model shows less homogeneity as compared to the NDRE model. X-axis represents the linear model, and y-axis represents the residuals of that model.
